# Supplementary material for: Morphological and ultrastructural investigation of the posterior atlanto-occipital membrane: Comparing children with Chiari malformation type I and controls
Source: PLoS One. 2024 Jan 16;19(1):e0296260. doi: 10.1371/journal.pone.0296260 (PMC10791003; doi:10.1371/journal.pone.0296260)
Supplement: Attachment — Submitted filename: PLoS One R2 Letter to Reviewers.docx [file pone.0296260.s003.docx]

November 16, 2023

Sagar Panthi, MBBS

Academic Editor

*PLOS ONE*

Dear Dr. Panthi:

Thank you for sending us the reviewers’ additional comments regarding our paper, "Morphological and ultrastructural investigation of the posterior atlanto-occipital membrane: comparing children with Chiari malformation type I and controls” (PONE-D-23-31525R1). We are pleased that the reviewers found the first round of revisions helpful, and in response to their comments, we have made the additional changes to the paper.

**RESPONSE TO REVIEWERS**

*Reviewer #1*

*The authors have addressed the reviewer comments.*

*One additional suggestion:
Recommend removing the final statement in conclusion regarding refutation of prior hypotheses. Because the abstract does not make reference to these hypotheses, it is difficult to understand what is meant by this comment.

Also, in the Conclusion section, while the refuted hypotheses have now at least been mentioned in the Discussion, this statement is probably a bit too strong given the provided data. Removing that clause about refutation would do nothing to weaken this report.*

Response:

We thank the reviewer and have made the suggested change and have removed the sentence about refutation of prior hypotheses from the conclusion section of the abstract and the conclusion of the manuscript itself.

We agree the suggested removal does nothing to weaken the report.

We thank the editor for the opportunity to revise the paper and believe that with the changes it is improved.

We hope it meets the standard for publication in PLoS One and look forward to your decision.

Sincerely,

Vijay M. Ravindra, MD, MSPH
